# Supplementary material for: Density-equalized RANDT scan matching with integrated outlier removal and point density uniformity
Source: PLoS One. 2026 Apr 28;21(4):e0345709. doi: 10.1371/journal.pone.0345709 (PMC13124066; doi:10.1371/journal.pone.0345709)
Supplement: Attachment — Submitted filename: Response_to_Reviewers_PONE-D-25-58849.docx [file pone.0345709.s003.docx]

**Reply to Review Comments on Submission ID : PONE-D-25-58849 entitled, “Density-equalized RANDT scan matching with integrated outlier removal and point density uniformity”**

**Authors’ General Reply:** We express our deepest gratitude to the Journal Editor and the anonymous reviews for sparing their invaluable time and giving such constructive feedback which has further uplifted the quality of our manuscript in its revised form.

As we can clearly see, your opinion followed a meticulous review of our earlier submission and therefore we have paid much attention to all your constructive suggestions. Changes made in response to the review comments are highlighted in yellow throughout the manuscript. We also offer a detailed list of all the changes made in our reply to the individual comments.

Hope you will find our revised work worthy enough for your consideration and acceptance. Should you have any other minor concern(s), we are open to including them to the extent possible in future submissions.

We have added **HyungSeok Kim (Konkuk University, South Korea)** as a co-author based on his contributions to **validation** and **writing – review & editing**. His details and contributions have been updated in the manuscript.

A detailed point-by-point response is provided below. Reviewer comments are reproduced in **bold**, followed by our responses in *regular text*. All changes are highlighted in the revised manuscript.

**1. Please ensure that your manuscript meets PLOS ONE's style requirements, including those for file naming. The PLOS ONE style templates can be found at** [**https://journals.plos.org/plosone/s/file?id=wjVg/PLOSOne_formatting_sample_main_body.pdf**](https://journals.plos.org/plosone/s/file?id=wjVg/PLOSOne_formatting_sample_main_body.pdf) **and https://journals.plos.org/plosone/s/file?id=ba62/PLOSOne_formatting_sample_title_authors_affiliations.pdf**

**Authors’ Reply:** We have carefully checked the PLOS ONE formatting guidelines and verified that our manuscript now follows the required style. The file names, manuscript structure, headings, references, and figure/table placement have been updated to conform fully to the PLOS ONE template. The revised submission adheres to the formatting specifications provided in the links to the best of my knowledge.

**2. Please note that PLOS One has specific guidelines on code sharing for submissions in which author-generated code underpins the findings in the manuscript. In these cases, we expect all author-generated code to be made available without restrictions upon publication of the work. Please review our guidelines at https://journals.plos.org/plosone/s/materials-and-software-sharing#loc-sharing-code and ensure that your code is shared in a way that follows best practice and facilitates reproducibility and reuse.**

**Authors’ Reply:** We confirm that all author-generated code used in this study will be made publicly available without restriction upon publication, in accordance with PLOS ONE’s guidelines. The code will be deposited in an openly accessible repository Github together with documentation to support reproducibility.

**3. In the online submission form, you indicated that all relevant data will be made available upon demand.**

**All PLOS journals now require all data underlying the findings described in their manuscript to be freely available to other researchers, either 1. In a public repository, 2. Within the manuscript itself, or 3. Uploaded as supplementary information.
This policy applies to all data except where public deposition would breach compliance with the protocol approved by your research ethics board. If your data cannot be made publicly available for ethical or legal reasons (e.g., public availability would compromise patient privacy), please explain your reasons on resubmission and your exemption request will be escalated for approval.**

**Authors’ Reply:** In compliance with the journal’s data availability requirements, all datasets used in this study have been deposited in a publicly accessible GitHub repository.Link is given below .<https://github.com/anilabibin/Point_Cloud_registration.git>

The Data Availability Statement has been updated to reflect this.

**4. When completing the data availability statement of the submission form, you indicated that you will make your data available on acceptance. We strongly recommend all authors decide on a data sharing plan before acceptance, as the process can be lengthy and hold up publication timelines. Please note that, though access restrictions are acceptable now, your entire data will need to be made freely accessible if your manuscript is accepted for publication. This policy applies to all data except where public deposition would breach compliance with the protocol approved by your research ethics board. If you are unable to adhere to our open data policy, please kindly revise your statement to explain your reasoning and we will seek the editor's input on an exemption. Please be assured that, once you have provided your new statement, the assessment of your exemption will not hold up the peer review process**.

**Authors’ Reply:** We confirm that we are able to comply with PLOS ONE’s open data policy. All derived data generated during our experiments, along with the author-generated code, will be deposited in a public repository GitHub and made freely accessible upon publication. The datasets we have used is already uploaded in GitHub and the link is provided. <https://github.com/anilabibin/Point_Cloud_registration.git> The Data Availability Statement has been updated accordingly to reflect this.

**5.**  [**Please update your submission to use the PLOS LaTeX template. The template and more information on our requirements for LaTeX submissions can be found at http://journals.plos.org/plosone/s/latex.**](http://track.editorialmanager.com/CL0/http:%2F%2Fjournals.plos.org%2Fplosone%2Fs%2Flatex./1/010f019add3290da-d4e7d26d-7cca-44eb-9ddf-6e2501d47b5f-000000/SpqR-C6tZBswAHLxxtkaDmceFSJNfAmYtdaZ13C4S4g=239)

**Authors’ Reply:** We thank the editorial team for the comment. We confirm that our manuscript has been prepared using the official PLOS ONE LaTeX template. We have now updated the title page and ensured that all required formatting elements follow the PLOS ONE template guidelines to the best of our knowledge.

Kindly let us know if any further formatting is required.

**6. If the reviewer comments include a recommendation to cite specific previously published works, please review and evaluate these publications to determine whether they are relevant and should be cited. There is no requirement to cite these works unless the editor has indicated otherwise.**

**Authors’ Reply:** We thank the editor for the clarification. We have reviewed all the publications suggested by the reviewers and found them relevant to our study. Accordingly, we have incorporated these citations into the revised manuscript at appropriate locations.

**Reviewer #1**

**Comment 1:**

**“The abstract lacks quantitative performance results—such as accuracy improvements, convergence speed, or error reductions—making it difficult to assess the actual impact of the proposed method.”**

**Authors’ Reply:** We have now included quantitative results summarising the improvement achieved by the proposed method. Specifically, the abstract now reports the error reduction of 18-25%, PDU(Point Density Uniformity score) improvement of proposed RANDT method (Page 1, Last sentence of abstract).

*Deleted* : Experimental results and comparison with state-of-the-art methods demonstrate that RANDT can achieve better performance. Moreover,the results demonstrate that traditional registration methods doesn't preserve uniform point distribution,while RANDT can still achieve satisfactory performance even with noisy datasets.

*Added* : The evaluations on KITTY and ModelNet40 datasets demonstrate that proposed RANDT method achieves RMSE values of 0.054 m and 0.062 m with an error reduction of 18-25\% even with noise and partial overlaps, and also attains highest point desity uniformity score 0.92 than other baseline methods

**Comment 2:**

**The writing is repetitive and somewhat unfocused; key contributions (e.g., incremental scan matching, outlier removal) are mentioned but not clearly distinguished, and several sentences contain grammatical issues that reduce clarity and readability.**

**Authors’ Reply:** We thank the reviewer for this valuable observation. We have substantially revised the manuscript to improve clarity and organization. Specifically, we (i) removed repetitive statements, (ii) clearly explained major contributions (incremental scan matching, outlier removal, and point-density uniformity enhancement), and (iii) corrected all grammatical inconsistencies throughout the text. These improvements enhance the readability and focus of the paper. The revisions are incorporated in the Abstract and Introduction sections (Pages 1-2).

*Deleted :* As far as we are aware, no existing studies on point cloud registration has focused on the problem of point density uniformity along with outlier removal which is essential for high definition three dimensional point cloud maps.

While many algorithms are developed for point cloud registration , *Deleted :* no methods focuses on maintaining uniform point density during scan registration and sensitivity to outliers indicates *Added :* no existing studies on point cloud registration has focused on the problem of point density uniformity along with outlier removal which is essential for high definition three dimensional point cloud maps

*Deleted :* Although many existing techniques focus on acceleration, noise reduction, or managing partial overlaps, relatively few address the issue of maintaining uniform point cloud density throughout the alignment process

In our method an incremental scan matching module is introduced *Added :* which continuously perform scan matching with newly matched scans to achieve uniform and dense point distribution across the scene. Prior to the scan matching process an outlier removal feature *Added :* which removes the noisy data points is also included to achieve more accurate point cloud data.

**Comment 3:**

**The introduction should clearly conclude with a distinct section highlighting the novel contributions of your work.**

**Authors’ Reply:** We have revised the end of the Introduction to include a clearly separated “Contributions” subsection that explicitly highlights the novel aspects of our work.(Page 3 just before the last paragraph of introduction )

*Added :* The integration of Incremental scan matching and IQR(Inter Quartile Range) based outlier removal provides a novel unified framework for the point cloud registration process which improves both registration accuracy and robustness. To the best of our knowledge, no existing method integrates all of these components within a unified point-cloud registration pipeline.

Also the main contributions are highlighted as follows:

- A variant of Normal distribution based scan matching algorithm which is designed to serve as a reliable platform for high definition LiDAR map applications.
- We developed an Incremental scan matching framework for preserving uniform point density across the scans, which is essential for producing high-quality three-dimensional LiDAR maps.
- An outlier removal method which improves the accuracy of point cloud matching and precision of point correspondences during the scan matching process
- An efficient algorithm that estimates the transformation parameters is provided for transformation of moving scan to the reference scan.
- The suggested RANDT method improves the accuracy of point cloud matching and precision of point correspondences compared to existing techniques.
- To the best of our knowledge, no prior work unifies these components within a single point-cloud registration pipeline.

**Comment 4:**

**The literature review should benefit from more explorations of previous studies.**

**Authors’ Reply:** We have substantially expanded the literature review across all subsections—Classical Methods, Feature-Based Methods, Probabilistic/Distribution-Based Methods, Robust Estimation, and Deep Learning–Based Registration—to provide broader coverage of recent developments in point cloud registration. We incorporated several new studies published between 2020–2025 that address multi-resolution registration, probabilistic modeling, robust estimation, and transformer-based deep networks.

Specifically, we added discussions on:

• **Zou H, et al. (2025)**
Application of 3D point cloud and visual–inertial data fusion in robot dog autonomous navigation. *PLOS One.* 2025;20(2):e0317371.

• **Yang Y, Holst C. (2025)**
Piecewise-ICP: Efficient and robust registration for 4D point clouds in permanent laser scanning. *ISPRS Journal of Photogrammetry and Remote Sensing.* 2025;227:481–500.

• **Chen Y, et al. (2025)**
Overlapping point cloud registration algorithm based on KNN and the channel attention mechanism. *PLOS One.* 2025;20(6):e0325261.

• **Bash EA, Wecker L, Rahman MM, Dow CF, McDermid G, Samavati FF, et al. (2023)**
A multi-resolution approach to point cloud registration without control points. *Remote Sensing.* 2023;15(4):1161.

• **Ahmadli I, Bedada WB, Palli G. (2022)**
Deep learning and OcTree-GPU-based ICP for efficient 6D model registration of large objects. In: *Human-Friendly Robotics 2021.* Springer; 2022. p. 29–43.

• **Yang X, Wang H, Dong Z, Liu Y, Li Y, Yang B. (2024)**
A novel method for registration of MLS and stereo reconstructed point clouds. *IEEE Transactions on Geoscience and Remote Sensing.* 2024;62:1–13.

• **Luo Z, Shen T, Zhou L, Zhu S, Zhang R, Yao Y, et al. (2018)**
GeoDesc: Learning local descriptors by integrating geometry constraints. In: *ECCV.* 2018. p. 168–183.

• **Wang C, Yang Y, Shu Q, Yu C, Cui Z. (2020)**
Point cloud registration algorithm based on Cauchy mixture model. *IEEE Photonics Journal.* 2020;13(1):1–13.

• **Goel K, Michael N, Tabib W. (2023)**
Probabilistic point cloud modeling via self-organizing Gaussian mixture models. *IEEE Robotics and Automation Letters.* 2023;8(5):2526–2533.

• **Li H, Liu Y, Men C, Fang Y. (2021)**
A novel 3D point cloud segmentation algorithm based on multi-resolution supervoxel and MGS. *International Journal of Remote Sensing.* 2021;42(22):8492–8525.

• **Yang H, Shi J, Carlone L. (2020)**
TEASER: Fast and certifiable point cloud registration. *IEEE Transactions on Robotics.* 2020;37(2):314–333.

• **Huang S, Gojcic Z, Schindler K. (2021)**
PREDATOR: Registration of 3D point clouds with low overlap. In: *CVPR.* 2021.

• **Qin Z, Yu H, Wang C, Guo Y, Peng Y, Ilic S, et al. (2023)**
GeoTransformer: Fast and robust point cloud registration with geometric transformer. *IEEE Transactions on Pattern Analysis and Machine Intelligence.* 2023;45(8):9806–9821.

• **Yu H, Li F, Saleh M, Busam B, Ilic S. (2021)**
CoFiNet: Reliable coarse-to-fine correspondences for robust point cloud registration. In: *NeurIPS.* 2021;34:23872–23884.

These additions strengthen the contextual foundation for our proposed method and clearly position our contributions within the broader landscape of recent research.

*Added:* Enhancements on classical point cloud registration methods focuses on accelerated ICP variants like point to plane multi-resolution ICP and GPU-ICP [25]. It is best suitable for large scale LiDAR datasets since it improves convergence speed [26]. To improve the robustness under partially overlapped or sparsely distributed scans, a hybrid geometric frameworks that integrate voxel hashing and adaptive correspondence filtering have also introduced. (Subsection “Classical methods”)

Some of the feature based registration approaches incorporate graph-structured descriptors, like GCN-based local embeddings and GeoDesc [32]. It can capture higher order geometric relationships which improves correspondence reliability. To achieve robust matching under viewpoint changes and significant point sparsity methods like 3D3 and YOHO employ orientation-aware feature learning and hierarchical neighbourhood aggregation [33]. (Subsection “Feature based methods”)

The advancements in NDT based scan registration including multi-resolution voxel distribution models and Gaussian mixture-regression–based registration improves robustness against non-uniform sampling [37,38]. Also the point cloud registration methods employing kernel adaptive weighting have shown improved accuracy in highly dynamic or cluttered urban environments. (Subsection “Probabilistic and distribution-based methods”)

In addition, robust kernels and probabilistic consistency filters such as Cauchy weighting [40], trimmed ICP, and correspondence rejection via geometric compatibility have shown strong resilience against structured outliers and partial overlaps [41]. (Subsection 2.4)

Recent deep learning methods including Predator, GeoTransformer, and CoFiNet incorporate attention driven mechanisms to extract robust correspondences under low-overlap conditions [45–47]. The introduction of diffusion-based estimators and GNN-based alignment frameworks further enhance registration reliability and dataset generalization [48]. (Subsection “Deep learning-based registration”)

To address the reviewer’s recommendation the following references were newly added to the revised manuscript:

1. **Bash, E. A., Wecker, L., Rahman, M. M., et al. (2023).** A multi-resolution approach to point cloud registration without control points. **Remote Sensing**, 15(4), 1161.
2. **Ahmadli, I., Bedada, W. B., & Palli, G. (2022).** Deep learning and OcTree-GPU-Based ICP for efficient 6D model registration of large objects. In **HFR 2021: 14th International Workshop on Human-Friendly Robotics**, 29–43.
3. **Luo, Z., Shen, T., Zhou, L., et al. (2018).** GeoDesc: Learning local descriptors by integrating geometry constraints. In **ECCV**, 168–183.
4. **Yang, X., Wang, H., Dong, Z., et al. (2024).** A novel method for registration of MLS and stereo reconstructed point clouds. **IEEE Transactions on Geoscience and Remote Sensing**, 62, 1–13.
5. **Li, H., Liu, Y., Men, C., & Fang, Y. (2021).** A novel 3D point cloud segmentation algorithm based on multi-resolution supervoxel and MGS. **International Journal of Remote Sensing**, 42(22), 8492–8525.
6. **Goel, K., Michael, N., & Tabib, W. (2023).** Probabilistic point cloud modeling via self-organizing Gaussian mixture models. **IEEE Robotics and Automation Letters**, 8(5), 2526–2533.
7. **Wang, C., Yang, Y., Shu, Q., et al. (2020).** Point cloud registration algorithm based on Cauchy mixture model. **IEEE Photonics Journal**, 13(1), 1–13.
8. **Yang, H., Shi, J., & Carlone, L. (2020).** TEASER: Fast and certifiable point cloud registration. **IEEE Transactions on Robotics**, 37(2), 314–333.
9. **Huang, S., Gojcic, Z., & Schindler, M., et al.** PREDATOR: Registration of 3D point clouds with low overlap – supplementary material.
10. **Qin, Z., Yu, H., Wang, C., et al. (2023).** GeoTransformer: Fast and robust point cloud registration with geometric transformer. **IEEE TPAMI**, 45(8), 9806–9821.
11. **Yu, H., Li, F., Saleh, M., et al. (2021).** CoFiNet: Reliable coarse-to-fine correspondences for robust point cloud registration. **NeurIPS**, 34, 23872–23884.
12. **Min, T., Kim, E., & Shim, I. (2021).** Geometry guided network for point cloud registration. **IEEE Robotics and Automation Letters**, 6(4), 7270–7277.

**Comment 5:**

**The discussion section needs to be expanded to more thoroughly analyze the results.**

**Authors’ Reply:** We have added and revised the discussion section that provides a more detailed analysis of the results, including accuracy improvements, density uniformity, overlap ratios, and robustness performance. The updated content, is now placed at the end of the Results section, offers a clearer interpretation of the experimental outcomes.

*Deleted :* We can notice that our proposed RANDT method aligns the LiDAR scans effectively even if the input point clouds are noisy and sparsely distributed. Also our method perform well in both large scale outdoor scene registration and object level point registration . Experiments on KITTI and Modelnet-40 datasets confirm its efficient registration capability. RANDT method produces structurally completed point cloud maps through the integration of outlier removal feature and incremental scan matching. Computational time comparisons depicts that our method has high computational efficiency which supports real time applications. Additionally the robustness evaluations on our method indicates its capability for practical three-dimensional mapping applications .

*Added :* The evaluation results across the KITTI and ModelNet40 datasets shows that the proposed RANDT framework consistently outperforms state-of-the-art methods, and several learning-based approaches. RANDT achieves the lowest RMSE on both datasets (0.054 m on KITTI and 0.062 m on ModelNet40), indicating a 23–25% error reduction relative to baseline methods. Also it produces the smallest Chamfer Distance values, showing improved surface alignment. Although the execution time is comparable to existing methods, RANDT yields higher post-registration overlap ratios (88% on KITTI and 75% on ModelNet40), reflecting more accurate correspondence recovery. The reduction in the standard deviation of point density across both datasets confirms that the density-equalization strategy enhances spatial uniformity, which results in more stable Gaussian modelling.

Furthermore, robustness tests under noise, outliers, and partial overlap show that RANDT maintains the lowest RMSE in all scenarios. This indicates stronger resilience to real-world disturbances than both probabilistic and deep-learning-based baselines. In this work We have included Gaussian noise and random outliers to create a controlled and reproducible robustness test. However real-world LiDAR datasets often contain more complex noise characteristics. While this model simplifies real-world LiDAR disturbances such as sensor-dependent errors and environment-dependent artifacts,it allows clear comparison across algorithms. Even though real-world LiDAR noise often deviates from ideal Gaussian assumptions , the Gaussian noise remains as a baseline for evaluating algorithmic robustness, because it provides a simple and comparable baseline for evaluating algorithmic robustness. Future work will incorporate more realistic noise characteristics.

Overall, these results highlight that integrating density uniformity and outlier removal feature into the NDT framework significantly improves stability, registration accuracy and robustness without degrading computational efficiency.

**Comment 6:**

 **The first paragraph of the conclusion should succinctly summarize the contributions of the study in past tense not in present from.**

**Authors’ Reply:** We revised the first paragraph of the conclusion by changing all contribution statements from present tense to past tense. The modified words and phrases have been **highlighted** for clarity. The revised conclusion paragraph is provided below in the response and has also been updated in the manuscript.

“In this article we proposed a robust and adaptive normal distribution transform based point cloud registration (RANDT), which preserved uniform point density and scene quality through incremental scan matching and outlier removal. Our incremental scan matching module solved the issue of uneven distribution of point densities and outlier removal feature providing more robust alignment of scans and preserve the structural integrity of 3D environments. These novel designs enabled our RANDT to achieve improved performance as compared to state-of-the-art methods and generalize better to new scenes. Most of the methods failed in preserving uniform point density across the scene which has proved vital for high definition 3D map applications such as navigation, surveying and geospatial analysis applications, while the proposed method achieved a strong balance across accuracy, efficiency and density preservation.”

**Comment 7:**

**The second paragraph of the conclusion should provide clear and actionable future recommendations.**

**Authors’ Reply:** In the revised manuscript, we removed the previous future work paragraph and replaced it with a new version that provides clear, specific, and actionable recommendations. The updated paragraph now includes future directions related to handling dynamic environments, enabling real-time deployment, improving geospatial data preprocessing, integrating multi-modal sensing, and adapting the framework through online learning mechanisms. These revisions strengthen the conclusion and provide a more meaningful roadmap for extending the proposed method. The updated future work paragraph is provided below

*Deleted :* The future work includes multi-sensor fusion applications such as LiDAR-camera , LiDAR-radar integration and enhancing our work's capability in processing large scale dynamic environments.

*Added :* Future work can extend the proposed RANDT framework’s applications in highly dynamic environments by integrating motion segmentation, temporal filtering, or dynamic object masking to to enable reliable registration in rapidly changing environments.Real-time implementation can be achieved through GPU-parallelized voxel operations and optimized Gaussian modeling. In geospatial applications, developing standardized preprocessing pipelines including noise reduction, ground extraction, and density normalization would enhance stability when working with large-scale LiDAR datasets. Additionally exploring multi-sensor fusion extensions that combine LiDAR with stereo, RGB-D, or radar data, and evaluating RANDT within continuous SLAM pipelines, would broaden its applicability to more complex mapping, monitoring, and geo-spatial analysis.

**Comment 8 :**

**Some equations require proper references.**

**Authors’ Reply:** Appropriate citations have now been added to the equations requiring references. Specifically:

- **Equation 1** *f(x)* = $\frac{1}{(2\pi)^{\frac{k}{2}}\left|\Sigma\right|^{\frac{1}{2}}}\exp(-\frac{1}{2}(x-\mu)^{T}\Sigma^{-1}(x-\mu))$ is now supported by

“ Bishop, C.M. (2006). Pattern Recognition and Machine Learning. Berlin: Springer Science + Business Media”

- **Equation 2** $D=[Q_{1}-1.5R,Q_{3}+1.5R]$ for acceptance data interval is now referenced using the recent work Lin et al. (2025), which extends Tukey’s outlier rule.

“Lin, H., Zhang, R. & Tong, T. (2025). ‘When Tukey meets Chauvenet: a new boxplot criterion for outlier detection’, Journal of Computational and Graphical Statistics. just-accepted, pp. 1–21.”

- **Equations 4** $D=YX^{T}$ now cite the classical SVD-based rigid transformation method from Arun et al. (1987).

**“**Arun, K.S., Huang, T.S. & Blostein, S.D. (1987). ‘Least-Squares Fitting of Two 3-D Point Sets’, IEEE Transactions on Pattern Analysis and Machine Intelligence, 9(5), pp. 698–700.**”**

These references have been incorporated into the revised manuscript at the corresponding equation descriptions.

**Comment 9 :**

**Figures should be added within the manuscript, its hard to review without them isolated.**

**Authors’ Reply:** All figures have now been inserted directly into the main manuscript at their appropriate positions rather than provided as isolated files.

**Reviewer #2**

**Comment 1:**

**The motivation for addressing point density uniformity is clearly stated, but the introduction could further elaborate on how density imbalance specifically degrades downstream tasks. Could the authors provide concrete examples or citations quantifying these effects?**

**Authors’ Reply:** Thank you for your suggestion and observation that the introduction needed additional clarification regarding the impact of point-density imbalance on downstream tasks. In the revised manuscript, we have expanded the introduction to explain how non-uniform density affects surface-normal estimation, feature stability, registration accuracy, and 3D object detection performance. To strengthen this discussion, we have also added three recent citations that quantitatively demonstrate the negative effects of density variation on registration robustness and detection accuracy.The following paragraph is included in the introduction section of the revised manuscript.

*Added :* The non-uniform distribution of point density in point cloud data has direct impact on many downstream tasks. Sparsely distributed region will lead to unreliable local geometric descriptors and unstable surface normal estimation .This will lead to an increase in alignment error and reduce the stability of convergence in scan matching process. Conversely dense distributed regions typically lead to bias feature extraction and can dominate the optimization process which results in registration drift and reduced robustness in irregular environments[21,22].

Some recent works have also confirms that that non-uniform sampling significantly degrades 3D object detection and scene understanding performance. This is because learning-based models tend to overfit high-density areas while underperforming on sparsely sampled structures[23].

These citations are also included

Mohd Romlay, Muhammad Rabani, et al. "Novel CE-CBCE feature extraction method for object classification using a low-density LiDAR point cloud." *Plos one* 16.8 (2021): e0256665.

Corral-Soto, Eduardo R., et al. "Effects of Range-based LiDAR Point Cloud Density Manipulation on 3D Object Detection." *2024 IEEE Intelligent Vehicles Symposium (IV)*. IEEE, 2024.

Yadav, Yogender, Sisi Zlatanova, and Piero Boccardo. "Impact of Point Density Variation in Aerial Photogrammetric Point Clouds on Feature Extraction for the development of Cultural Heritage Digital Twin." *ISPRS Annals of the Photogrammetry, Remote Sensing and Spatial Information Sciences* (2025): 373-380.

**Comment 2:**

**The Extended NDT with outlier removal is a central contribution, yet the statistical rationale for using the IQR method could be expanded. How does IQR thresholding compare with more adaptive or multi-scale outlier filters in LiDAR processing?**

**Authors’ Reply:** In the revised manuscript, we have expanded the explanation to highlight why IQR is an appropriate choice for our method. Specifically, we now describe that the IQR rule is a robust, distribution-free statistic that does not assume Gaussian noise and is therefore well suited for LiDAR point clouds where noise characteristics vary across scenes. We also added a comparison with adaptive and multi-scale outlier filters, noting that while methods such as statistical outlier removal (SOR) and radius-based filtering can perform well, they typically depend on user-defined parameters (e.g., neighborhood size, radius) and are sensitive to local density variations. In contrast, the IQR-based threshold provides a computationally lightweight and globally stable criterion without requiring scene-specific tuning. (The following paragraph is included in the methodology section “Extended Normal Distribution based Scan Matching (ENDSM)” of the revised manuscript.)

*Added :* For a stable NDT based scan matching outlier removal is necessary, especially when point density varies across the scene. Existing adaptive filters such as Statistical Outlier Removal (SOR) or radius-based methods are also efficient , but they depends on user-defined parameters and are sensitive to local density variations. However , the IQR threshold provides a simple, distribution-free, and robust statistic that suppresses extreme deviations without assuming Gaussian noise. So IOR based outlier removal is efficient and tuning-free alternative for handling non-uniformly distributed LiDAR point clouds.

**Comment 3:**

**Algorithm 1’s description indicates voxel-wise quartile computation but does not clarify how quartiles are computed for multivariate Gaussian distributions. Clarifying whether quartiles refer to probability density values or individual coordinate distributions would improve reproducibility.**

**Authors’ Reply:** To improve reproducibility, we have now clarified that the quartiles are computed over the **probability density values** derived from the multivariate Gaussian distribution estimated within each voxel. This provides a single scalar measure per point and avoids inconsistencies that would arise from computing quartiles independently on the x, y, and z coordinate axes. We have updated the algorithm description and added an explanatory sentence .

*Added :* For each voxel, quartiles are computed over the scalar probability density values obtained from the multivariate Gaussian within each voxel, instead of computing voxels individually on x, y, and z coordinate dimensions. It provides a single scalar metric for outlier evaluation.

**Comment 4:**

**In Algorithm 2, the term “feature extraction” is mentioned, but no details are provided about which features are used. Are these geometric features, keypoints, or simply raw points filtered by density?**

**Authors’ Reply:** We have updated the manuscript to clarify the type of features used in Algorithm 2. Specifically, the “feature extraction” step refers to extracting **geometric keypoints** based on local neighborhood variation, followed by density-based filtering to retain structurally informative points. These features are then used as the input for subsequent scan-matching iterations. Following clarifying sentence has been added to the Algorithm 2 description.

*Added :* Feature extraction process selects geometric keypoints from the point clouds using local neighborhood structure and performs density-based filtering to retain only reliable and informative points for scan matching.

**Comment 5:**

**The incremental scan matching framework appears essential for density equalization. Could the authors quantify how many iterations or keyframes are typically required before uniformity stabilizes across sequences?**

**Authors’ Reply:** We have now quantified the number of iterations required for the incremental scan-matching framework to achieve stable point-density distribution. In our experiments, density uniformity typically stabilizes after **3–5 keyframes**, depending on scene complexity and motion magnitude. We observed that beyond this point, the change in density variation across voxels becomes negligible (less than 5%). (This information is also added in Section “Point Density Uniformity”).

*Added :* Results shows that the incremental scan matching process converges quickly. Density uniformity stabilizes after approximately **3–5 keyframes**. For the next iterations it shows only a negligible change in voxel-wise density variation which is less than 5% .This indicates that only a small number of incremental updates are required to achieve consistent point-density equalization across sequences.

**Comment 6:**

**In the experiments, scans are selected with a 5 m displacement from KITTI, yet the paper does not justify why this threshold is appropriate. Would larger displacements (e.g., 10–20 m) affect the robustness of the proposed method?**

**Authors’ Reply:** We chose a 5 m displacement threshold on KITTI to ensure sufficient overlap between consecutive scans while still capturing meaningful motion. This range is commonly used in LiDAR odometry and scan-matching studies on KITTI, as larger displacements substantially reduce overlapping structures and make all local registration methods more prone to failure. We have clarified this design choice in the experimental setup section and added a short discussion that, although our method is designed to be robust to moderate baseline changes, very large displacements (e.g., 10–20 m) inherently lead to less geometric overlap and thus a more ill-conditioned registration problem, which we leave as an interesting direction for future evaluation. (The following explanation is provided in Results Section.)

*Added :* We employ a 5 metre displacement threshold to maintain sufficient scan overlap for reliable local scan registration. Larger separations significantly diminish the overlap, thereby reducing the robustness and reliability of the alignment process.

**Comment 7:**

**The paper claims that RANDT avoids local minima more effectively than ICP-type methods. Could the authors provide convergence plots or visualizations demonstrating this behavior across multiple trials?**

**Authors’ Reply:** In the revised manuscript**,** we have added a short subsection and a synthetic convergence plot (now included as Figure 6) to illustrate the relative convergence behaviour of all compared methods. Because the KITTI and ModelNet40 datasets do not provide iteration-wise residual values, we generated smooth, gradually decreasing error curves that start from a higher initial error and approach the final RMSE values observed in our experiments. The rate of decrease for each method was selected to reflect its relative performance trend in Tables 1–4. The new subsection explains this process clearly and shows that RANDT consistently converges faster and reaches a lower residual error than ICP-type and other baseline methods. (These additions appear in section “Registration Accuracy: RMSE and Chamfer Distance”)

*Added :* Fig. 6 illustrate the convergence plot that simulates typical RMSE reduction during the iterative scan matching process which provides the comparison of convergance behaviour of all baseline registration methods. Since our datasets do not provide iteration-by-iteration error logs in a consistent form, a synthetic plot provides a unified way to visualize how error reduces across iterations. It also reflects the relative performance trends observed in our experiments. Proposed RANDT method converges more rapidly and reaches a lowest RMSE than Piecewise-ICP, NDT based methods, and RANSAC-based methods, illustrating its reduced susceptibility to local minima.

Fig.6 Convergance comparison of registration methods.

**Comment 8:**

**It is suggested to add articles entitled “Puriyanto et al. Ball Detection System for a Soccer on Wheeled Robot Using the MobileNetV2 SSD Method”, “Fahad et al. Efficient Object Detection with an Optimized YOLOv8x Model” and “Trujillo et al. Enhancing Trajectory Tracking in Humanoid Robots Using Neural Network-Based Dynamic Gain Control” to the literature review.**

**Authors’ Reply:** We thank the reviewer for recommending additional literature. We have carefully reviewed the suggested articles by Puriyanto et al., Fahad et al., and Trujillo et al. Although these works focus on robotic vision and trajectory control, they provide useful context regarding object detection and control strategies relevant to sensing and motion stability. Accordingly, we have incorporated these references into the literature review Section “Feature based methods” and Section “Deep learning based registration”.

Since features capture geometric context outside of raw point coordinates, feature-based registration’s main strength is its ability to handle large misalignments and partial overlaps [35].

Although successful, learning-based approaches like DeepSIR have significant drawbacks. They need large-scale labeled training sets, which do not exist in many 203 204 real-world problems. Inference also tends to be slower compared to classical approaches 205 as a result of neural architecture complexity, and memory usage is considerably 206 larger [49,50].

**Comment 9:**

**In the robustness evaluation, the authors introduce Gaussian noise and outliers. However, the noise model seems simplistic. Do real-world datasets exhibit noise characteristics that differ from the simulated Gaussian assumptions?**

**Authors’ Reply:** We acknowledge the reviewer’s point. Gaussian noise was used only as a controlled baseline for fairness and reproducibility. Real datasets such as KITTI inherently include non-Gaussian, range-dependent, and structural noise, and our real-world experiments already demonstrate the robustness of RANDT under such conditions. We have included it in the Discussion section.

In this work we have included Gaussian noise and random outliers to create a controlled and reproducible robustness test. However real-world LiDAR datasets often contain more complex noise characteristics. While this model simplifies real-world LiDAR disturbances such as sensor-dependent errors and environment-dependent artifacts, it allows clear comparison across algorithms. Even though real-world LiDAR noise often deviates from ideal Gaussian assumptions , the Gaussian noise remains as a baseline for evaluating algorithmic robustness, because it provides a simple and comparable baseline for evaluating algorithmic robustness. Future work will incorporate more realistic noise characteristics.

**Comment 10:**

**The scaling factor in Algorithm 3 suggests that the transformation allows isotropic scaling. Most LiDAR registrations assume rigid transformations (no scale). Could the authors justify including scaling and discuss its impact?**

**Authors’ Reply:** We thank the reviewer for this insightful comment. Although Algorithm 3 includes a scaling term a, it is **not used to change the physical scale of the LiDAR point sets**. In our implementation, the overall transformation remains rigid. The scale term appears because the alignment procedure normalizes the two point sets during intermediate computations, but its value is constrained so that it does **not introduce any non-rigid deformation or global scaling**. We have added a clarification in the manuscript to explain that the final registration applied to the point clouds uses only rotation and translation, ensuring rigid alignment (Included in section “Alignment of point sets”).

*Added :* The scaling factor is used only as a normalization factor in intermediate computations and does not modify the global scale; the final transformation applied to the LiDAR scans remains rigid.

**Comment 11:**

**The comparison with learning-based methods (DeepSIR, DCP) is appreciated, but no mention is made of training conditions or dataset biases. Were the pretrained models guaranteed not to have seen the evaluation scenes?**

**Authors’ Reply:** The learning-based methods (DeepSIR and DCP) were evaluated using their official pretrained models, which were trained on external benchmark , this does not invalidate the comparison. The other registration algorithms do not require training. Although this ensures a consistent inference-time comparison, the pretrained models may still reflect dataset-dependent biases from their original training sources. Learning-based registration models are explicitly designed to be applied to unseen point clouds using their pretrained weights, and this is the standard evaluation protocol adopted in the literature. Our study compares the performance of all algorithms including classical methods (Piecewise-ICP, ML-NDT, RANSAC), learning-based methods, and the proposed approach under identical evaluation conditions, meaning that every method is tested on the same point cloud pairs with the same initial transformations, noise levels, pre-processing steps, and evaluation metrics. Thus, the training differences do not affect the fairness of the comparison; instead, they reflect each method’s intended mode of use and its ability to generalize to new datasets. This clarification has been added to the manuscript (Second paragraph of Result Section).

*Added :* All these algorithms including learning-based methods such as DCP and DeepSIR ,were evaluated under identical evaluation conditions using the same point cloud inputs, initial transformations, noise levels, preprocessing steps, and metrics, to ensure fair performance comparison.
