# Supplementary material for: Density-equalized RANDT scan matching with integrated outlier removal and point density uniformity
Source: PLoS One. 2026 Apr 28;21(4):e0345709. doi: 10.1371/journal.pone.0345709 (PMC13124066; doi:10.1371/journal.pone.0345709)
Supplement: Attachment — Submitted filename: Response to reviewer _MinorRevision_PONE-D-25-58849.docx [file pone.0345709.s004.docx]

**Reply to Review Comments on Submission ID : PONE-D-25-58849 entitled, “Density-equalized RANDT scan matching with integrated outlier removal and point density uniformity”**

**Authors’ General Reply:** We express our deepest gratitude to the Journal Editor and the anonymous reviewers for sparing their invaluable time and giving such constructive feedback which has further uplifted the quality of our manuscript in its revised form.

As we can clearly see, your opinion followed a meticulous review of our earlier submission and therefore we have given much attention to all your constructive suggestions. Changes made in response to the review comments are highlighted in yellow throughout the manuscript. We also offer a detailed list of all the changes made in our reply to the individual comments.

Hope you will find our revised work worthy enough for your consideration and acceptance. Should you have any other minor concern(s), we are open to including them to the extent possible in future submissions.

A detailed point-by-point response is provided below. Reviewer comments are reproduced in **bold**, followed by our responses in *regular text*. All changes are highlighted in the revised manuscript.

**Reviewer #1:**

**Well responded, however, remove extra information from the abstract now please. it is a but extra in content.**

Authors Response : Thank you. The abstract has been shortened by removing the extra explanatory sentences and simplifying the description of the registration requirement.

*Deleted :* For constructing high definition three-dimensional environmental models with focus on reducing noise disturbances and preserving uniform point density an efficient point cloud scan matching or registration is required.

*Added :* Accurate registration is required to reduce noise and maintain uniform point density.

*Deleted :* Outliers or noises also degrade the accuracy of the maps. This leads to variations in the density of point distribution throughout the matched point cloud scan.

(From Abstract)

**Reviewer #2:**

**The article has been revised very well, so I would suggest to accept in its present form.**

 Authors Response : Thank you

**Reviewer #3:**

**Figures 4, 5, and 8 effectively illustrate the registration results. However, in Figure 8 (point density uniformity comparison), consider adding:

A colour scale legend showing what the colours represent (point density values)

Quantitative labels on the visualizations showing actual density variance values
This would make the visual comparison more interpretable for readers.**

 Authors Response : Thank you for the helpful suggestion. We have revised Figure 8 to include a **color legend** clarifying the meaning of the bar colors, which improves the interpretability of the point density variance comparison.

**Minor editorial suggestions**

**Comment 1**

**Page 1, Abstract: “desity uniformity” → “density uniformity” (typo in line 17 of abstract).**

Authors Response : Thank you for pointing this out. The typo has been corrected.

**Comment 2**

**Page 9, Algorithm 2, Line 4: Consider rephrasing “for all Reference scan and Moving scan do” to “for each scan pair do” for clarity.**

Authors Response : Thank you. The wording has been updated as suggested for clarity.

**Comment 3**

**Page 22, Line 534: “solved the issue” → “addresses the issue” (to maintain consistency with present tense used elsewhere in conclusions).**

Authors Response : Thank you. The wording has been updated as suggested

**Comment 4**

**References: Verify that all in-text citations match the reference list. I noticed some formatting inconsistencies (e.g., reference 28 vs 29).**

Authors Response : Thank you. All in-text citations now match the reference list, and the references have been reformatted for consistency according to the journal style.

**Reviewer #4**

**Comment 1**

**Grammar:
There are many issues related to punctuation and spacing. There should never be a space before a period or comma, and there should always be a space at the beginning of a sentence. The first example is in the abstract: "Transform (RANDT) .In"**

Authors Response : Thank you for pointing this out. We have corrected the punctuation and spacing issues throughout the manuscript, including the example in the abstract.

**Comment 2**

**There are also some other grammar and spelling issues. Some examples include "overcoome" and "It offer"**

Authors Response : Thank you for highlighting the grammar and spelling issues. We have carefully proofread the manuscript and corrected these errors throughout the text.

**Comment 3**

**One of the author names on the "Author Contributions" list on p. 22 is spelled differently than in the author list on p. 1**

Authors Response : Thank you for noting this error. The author’s name was misspelled in the Author Contributions section on page 22 and has now been corrected to “Umashankar Subramaniam” to match the author list.

**Comment 4**

**Algorithm Clarity: Some aspects of the algorithm description remain unclear.
 One of the most important algorithmic clarity issues involves equations (1) and (2). A one-dimensional distribution has clearly defined quartiles, but the definition of Q is not clear for a multi-dimensional distribution; it is possible (again not clear) that the quartiles were derived from f(x) rather than the samples x, but this again does not make sense because f(x) is one sided and the description in (2) assumes a two sided distribution of points.**

Authors Response : Thank you for pointing out this lack of clarity. We have revised the manuscript to clarify that quartiles for each sample and rewritten that the computed values are from a scalar Mahalanobis distance, and quartiles are derived from the distribution of these distance values across all samples. This explanation has been added in the text before Eq. (2).

*Added:* “Algorithm 1 introduces this extension, which is the Extented Normal Distribution Scan Matching(E-NDSM). The input reference scan is divided into uniform voxels in the 251 first step. The set of points in a voxel is compactly represented by its mean vector and 252 covariance matrix, creating a multivariate Gaussian distribution, rather than matching 253 every point within each voxel. For each voxel, quartiles are computed over the scalar 250 254 probability density values obtained from the multivariate Gaussian within each voxel, instead of computing voxels individually on x, y, and z coordinate dimensions. Hence it 256 provides a single scalar metric for outlier evaluation. Since the pair (µ,Σ) can statistically describe the entire voxel, this lowers the difficulty of correspondence estimation [58]. Although the data are multi-dimensional, quartile-based outlier removal requires scalar values. Therefore, for each sample *x*, we compute a scalar distance measure from the Gaussian model using the Mahalanobis distance. Quartiles *Q1* and *Q3* are then computed from the distribution of these distance values across all samples.”

**Comment 5**

**With regard to the pseudocode for Algorithm 2, the feature extraction step (line 1) has now been described in the text with one added sentence, but the methodology is imprecise. Is there a paper you can cite that describes the "density-based filtering" method you've applied?**

Authors Response : Thank you for the comment. We have clarified the feature extraction step in Algorithm 2 and added a citation to relevant literature describing a density-based point cloud filtering method (Deng et al., 2022) that motivates our approach. The reference is provided below.

*Added :* The density-based filtering step used in feature extraction is inspired by density-clustering-based algorithm proposed by Deng et al. [60] which efficiently separates ground, object, and noise like meaningful points in LiDAR data using density information .

[60] Deng, Xingsheng, Guo Tang, and Qingyang Wang. "A novel fast classification filtering algorithm for LiDAR point clouds based on small grid density clustering." *Geodesy and Geodynamics* 13.1 (2022): 38-49.

**Comment 6**

**A second regarding Algorithm 2 is the definition of the "get" and "set" operations (e.g. in lines 5 and 6). It seems to me these can't simply be replacement operations (e.g. arrow meaning "becomes equal to"), as I thought that the reference frame needed to be a fusion of multiple raw frames in order to implement the equalization scheme. Bottom line, I'm a strong believer in point equalization in lidar scan matching ... but I still have no idea how this aspect of your algorithm was implemented.**

Authors Response : Thank you for this helpful comment. We have clarified that in Algorithm 2 the “get” operation retrieves the current fused reference model (keyframe) accumulated from previously aligned scans, while the “set” operation performs an incremental fusion of features from the newly aligned scan into this model. These operations therefore represent access and update of a global reference representation rather than simple frame replacement.

*Added :* The ‘Get’ operation retrieves the current fused reference model (keyframe), which has been incrementally constructed from previously aligned scans, while the ‘Set’ operation updates this model by integrating features from the newly aligned scan.

**Comment 7**

**In describing the results, a missing detail is a statement of the source of the ground truth. For KITTI, was GPS/INS used as ground truth to compute RMSE?**

Authors Response : Thank you for pointing this out. We have added a clarification in the manuscript stating that the ground-truth poses for the KITTI dataset are provided by the benchmark and were obtained using a high-precision GPS/INS system, which we used to compute the RMSE.

*Added :* For the KITTI dataset, the ground truth is provided by a GPS/INS system, specifically a high-grade RTK GPS tightly coupled with an IMU (OXTS) mounted on the vehicle.
